# Chronological Changes in Japanese Physicians' Attitude and Behavior Concerning Relationships with Pharmaceutical Representatives: A Qualitative Study

**DOI:** 10.1371/journal.pone.0106586

**Published:** 2014-09-19

**Authors:** Sayaka Saito, Kei Mukohara, Yasushi Miyata

**Affiliations:** 1 Department of Primary Care and Medical Education, Graduate School of Comprehensive Human Sciences, University of Tsukuba, Ibaraki, Japan; 2 Department of General Medicine, National Hospital Organization Nagasaki Medical Center, Nagasaki, Japan; 3 Department of General Internal Medicine, Rumoi Municipal Hospital, Hokkaido, Japan; ISPO, Italy

## Abstract

**Background:**

Recent qualitative studies indicated that physicians interact with pharmaceutical representatives depending on the relative weight of the benefits to the risks and are also influenced by a variety of experiences and circumstances. However, these studies do not provide enough information about if, when, how and why their attitudes and behaviors change over time.

**Methods and Findings:**

A qualitative study using semi-structured face-to-face individual interviews was conducted on 9 Japanese physicians who attended a symposium on conflicts of interest held in Tokyo. Interviews were designed to explore chronological changes in individual physicians' attitude and behavior concerning relationships with pharmaceutical representatives and factors affecting such changes. Their early interaction with pharmaceutical representatives was passive as physicians were not explicitly aware of the meaning of such interaction. They began to think on their own about how to interact with pharmaceutical representatives as they progressed in their careers. Their attitude toward pharmaceutical representatives changed over time. Factors affecting attitudinal change included work environment (local regulations and job position), role models, views of patients and the public, acquisition of skills in information seeking and evidence-based medicine, and learning about the concepts of professionalism and conflict of interest. However, the change in attitude was not necessarily followed by behavioral change, apparently due to rationalization and conformity to social norms.

**Conclusions:**

Physicians' attitudes toward relationships with pharmaceutical representatives changed over time and factors affecting such changes were various. Paying attention to these factors and creating new social norms may be both necessary to produce change in behavior consistent with change in attitude.

## Introduction

The most important professional responsibility of physicians is serving patients' best interest. However, this primary interest is sometimes undermined by secondary interest. One of the most notable concerns about conflict of interest has been the relationship between physicians and the pharmaceutical industry. In several countries, pharmaceutical representatives (PRs) give physicians a variety of gifts, information about products, and financial support for educational events, all with the aim of promoting products [1]–[6]. A gift-giving relationship between physicians and the pharmaceutical industry is ethically problematic for the following reasons: gifts cost money [7]; acceptance of gifts possibly erodes the faith of a patient in his or her doctor [8], [9]; gifts establish the obligation to respond possibly resulting in influence on physicians' decisions in patient care [10], [11]. Some studies have reported associations between exposure to information directly from the pharmaceutical industry and higher frequency, lower quality, or higher cost of prescribing, and others have not found such associations [12]. There is little systematic research on the impact of financial support for continuing education for physicians, although there are some reports of potential bias in favor of the pharmaceutical industry [13]–[15]

Remedies to control conflict of interest range from individual discretion to collective regulations including legal action [16], [17]. In the past decade, guidelines and recommendations were developed by an expert panel, professional societies, accrediting bodies, the pharmaceutical industry associations and the government in the United States [18]–[22]. In Japan, physicians working for public hospitals are prohibited from accepting kickbacks [23]. The Code of Pharmaceutical Research and Manufacturers of America in 2009 was translated and adopted by the Japan Pharmaceutical Manufacturer Association in 2011 [24]. However, at present, no professional societies in Japan have issued guidelines on physicians' relationships with pharmaceutical representatives in the context of clinical practice and medical education. After introduction of those guidelines and regulations in the United States, physician-industry relationships were nationally surveyed in 2009 and still high proportion (84%) of respondents had some type of interactions with PRs, although that was lower than that in 2004 (94%) [25]. In a questionnaire survey of 515 obstetricians and gynecologists in the United States, having read the guidelines was not associated with less reliance on PRs [26] These results indicate that the guidelines and regulations are partly effective but limited in reducing the number of physicians who engage in relationships with the pharmaceutical industry. In a survey of US family medicine residents, those from restricted programs reported fewer interactions with PRs, perceived less benefit from interactions with PRs, and rated gifts as less appropriate [27]. The previous national survey of Japanese physicians also showed an association between presence of local rules and less physician interaction with PRs [5]. It was also shown that attending medical schools with restricting policy was associated with reduced prescribing of newly marketed psychotropic medications [28]. These findings indicate that institutional rules and regulations may be more effective than general guidelines and recommendations in affecting physicians' attitude and behavior toward PRs.

The effectiveness of educational interventions aimed at helping individual physicians judiciously manage the conflict of interest has been inconclusive [29], [30].

Some qualitative studies investigated the reasons why physicians continue to interact with PRs regardless of the ethical implications. Physicians believe that the information from PRs is useful and that they can adequately evaluate the information without negative influence [31], [32]. Another reason for meeting with PRs has been reported to be as a part of their social contracts [31]. In one qualitative study, physicians were categorized into three types (avoiders, ambivalent engagers, and confident engagers) according to their attitude toward relationships with PRs [33]. In that study, physicians determine how to interact with PRs according to the perceived benefits and risks of the relationship, which were differently weighed from person to person. These findings suggest most physicians regularly interact with PRs and various factors influence such interactions. However, they do not provide enough information about if, when, how and why their attitudes and behaviors change over time.

To better understand how and why physicians interact with PRs over time, we conducted in-depth individual interviews with physicians focusing on chronological changes in their attitude and behavior concerning relationships with PRs and factors affecting such changes.

## Methods

### Participants

Participants were recruited using purposive sampling to select those who were able to reflect on themselves with some knowledge of conflict of interest. In a symposium titled “Conflicts of Interest in Clinical Practice and Medical Education” supported by the Japanese Society of Medical Education and held in Tokyo on December 8, 2012, attendees were notified of the purpose and nature of this study. From them, nine physicians gave written consent. All participants were contacted via email by KM and interviews were scheduled individually. The study protocol was approved by the Institutional Review Board of Hokkaido University Hospital.

### Data Collection

KM conducted face-to-face in-depth individual interviews with 9 physicians between January and March 2013. Three interview sessions were observed by SS. The interviews were semi-structured and designed to elicit each participant's chronological changes in his or her attitudes and behavior concerning relationships with PRs and factors affecting the changes. Interviews usually lasted 30 to 60 minutes. With the permission of interviewees, all interviews were digitally recorded and transcribed verbatim.

### Data Analysis

The transcripts were analyzed according to the “Steps for Coding and Theorization” (SCAT) method [34]–[38], which is a qualitative data analysis technique for generative coding and theorization. It consists of four steps of coding in which a researcher edits segmented text, filling in the following 4 separate columns: (1) focused words from the segmented text, (2) words outside of the text that are replaceable with the words from step (1), (3) words which explain the words in step (1) and (2), and (4) themes and constructs. Then, themes and constructs coded in step (4) are woven together to write a storyline and offer theories. We chose SCAT for its explicit process of analysis and for its efficiency and validity of theorization from relatively small scale data. All of the transcribed texts were included in the analysis and the whole process of analysis was recorded on charts which could be reviewed by other researchers. All the steps of initial coding and theorization developed by SS were reviewed and confirmed by KM and YM.

## Results

A total of nine physicians (eight men and one woman) were interviewed. Five physicians were specialized in general medicine, two in family medicine, one in respiratory medicine, and one in infectious disease. Their years since graduation ranged from 10 to 31. Six (#1, 3, 4, 5, 7, 8) out of nine participants had graduated more than 20 years earlier. Six physicians (#1, 2, 4, 5, 7, 9) reported having interacted with PRs throughout their career. After the initial period of exposure, physicians #3 and #8 had a period in which they had no interaction with PRs but resumed the relationship after their workplaces changed. Physician #6 had contact with PRs in his medical school but never saw PRs after he began practice because of local regulations.

Three stages of chronological change in attitude and behavior were derived from a collective narrative of 9 physicians: stage 1, early interaction (pre-contemplation); stage 2, change in attitude (contemplation); stage 3, change in behavior (action) (Table 1).

**Table 1:** Chronological changes in Japanese physicians' attitude and behavior toward interaction with pharmaceutical representatives.

Stage 1: Early interaction (pre-contemplation)	Physicians passively interacted with pharmaceutical representatives by merely joining their superiors.
Stage 2: Change in attitude (contemplation)	They began to think about how they should interact with PRs. Their attitude changed over time. Factors affecting attitudinal changes include: work environment, role models, views of patients and the public, acquisition of skills in information seeking and evidence-based medicine, and learning about the concepts of professionalism and conflict of interest.
Stage 3: Change in behavior (action)	Change in attitude was not always followed by behavioral change because of rationalization and conformity to social norms.

### Stage 1: Early interaction (pre-contemplation)

First contacts of participants with PRs were always arranged by their superiors. Interactions in their early years were passive as they joined their superiors participating in sponsored lectures at or outside workplaces and in recreational activities. They were not explicitly aware of the meaning of such interaction. Six physicians (#1, 4, 5, 6, 7, 8) took for granted interacting with PRs and did not think much about the legitimacy of the interaction. Three physicians (#2, 3, 9) remembered having felt uncomfortable interacting with PRs at that time.

*When I was in a clinical clerkship at medical school, we were given a lunch box and ate it while listening to a presentation by a company. We were also given gifts such as notebooks, notepads, and pens. I took it as a matter of course, and when there was no gift, I felt I missed out. … It was an ordinary thing. (physician #6)*

*My first interaction was outside the hospital when I was a medical student. I sometimes had a snack at evening conferences. So, I took it for granted. At that time, I thought it was common practice…. When I was a trainee, I did not think much about it because I thought that attending physicians decided how to interact with PRs anyway. (#8)*

*I saw the real world then. My superior seemed to take it for granted. When they took me out, there were often PRs with us. I simply sat with them and didn't think about whether it was right or wrong. I thought it was natural for physicians-in-training to get used to that kind of interaction because it was common among their superiors. (#2)*

### Stage 2: Change in attitude (contemplation)

As they progressed in their careers, they became conscious of their attitude toward PRs in their own ways and began to think about how they should interact with them. Their attitude toward relationship with PRs changed over time. Factors promoting changes toward critical attitude included their work environment (local regulations and job position), role models, views of patients and the public, acquisition of skills in information seeking and evidence-based medicine (EBM), and learning about the concepts of professionalism and conflict of interest.

#### Factors affecting attitudinal change

- Work environment: local regulations and job position

Six physicians (#3, 4, 5, 6, 8, 9) changed the way they interacted with PRs according to the rules of the workplace.

*After I was transferred to a national hospital, there were relatively strict rules saying that PRs' visits had to be after a certain hour and receiving meals from the industry was prohibited. I didn't have to restrain myself from interacting with PRs. I didn't do that because I was far away from it at that time. (#4)*

*When I was a junior resident, according to hospital policy, there were no educational meetings sponsored by the industry and no acceptance of gifts. Then, I didn't accept gifts, not even pens, nor did I listen to presentations by PRs. Colleagues at other hospitals were having interactions with PRs and I felt a bit disadvantaged. (#6)*

*I started to work at a public hospital in the countryside three years after graduation. In that public hospital, there already were strict rules prohibiting entertainment by pharmaceutical companies and presentations by PRs.… But, for me, it did not matter. I did not care much about the absence of such entertainment or meals. (#8)*

When a physician (#5) was a resident, he prescribed drugs as instructed by his attending physicians and did not have a chance to choose drugs on his own. Once he started to practice independently, he had to choose drugs by himself from a long list. Also, PRs came to see him in order to promote their drugs and he had to actively think about how to interact with them.

*PRs didn't have direct contact with me when I was a trainee at the university hospital. When I started to work at a community hospital, I had to decide everything by myself. … Then PRs came to see me, and I noticed. …Thereafter, I felt free to ask them for information about new medication. I even asked them to obtain scientific articles for me. (#5)*

For five physicians (#1, 4, 5, 6, 7), assuming the position of an educator or a leader in an institution was an opportunity to actively think about the legitimacy of interaction with PRs.

*When I got a position as an assistant professor in a university, I had to teach residents how to use medications. At that time, two different companies sold amlodipine (antihypertensive medication) and two PRs from the different companies came to see me.… I had to listen to both of them. I thought I should not favor one over the other and began to wonder what I should do in that kind of situation. (#1)*

*I thought I should behave properly because I was the director at the hospital. (#1)*

*I started to think seriously about the issue when I started to work at a postgraduate clinical training center about ten years ago. I was working on education for students and residents. … Residents were exposed to pens, some novelty goods, and lunch boxes at seminars. I began to think about how to teach about physician-PR relationships… (#4)*

*In the position of educating young physicians, I thought that it was not OK to always ask PRs for something. (#5)*

The attitudes of a physician (#7) toward current interaction between PRs and trainees were more critical than his attitude to his own interaction with PRs in the past.

*When I think about how residents should interact with PRs, because of my own vivid memory of my first interaction with PRs, I would not accept a situation for residents such as playing golf with PRs on the weekday (like my first interaction). (#7)*

- Role models

The way colleagues interacted with PRs provoked discomfort and critical attitudes among two physicians (#2, 5).

*In a hospital that employed only a few doctors, the director of the hospital had power over most matters. We often experienced an abrupt change in the drug formulary at an ambulatory clinic when we worked. Some of us wondered what had happened. Then, I heard that there had been entertainment for the hospital staff the week before. (#2)*

*It was habitual for one of my superiors to be driven home by PRs and stop for a drink with PRs on the way. He often invited me to a meal and then one specific PR always accompanied us. I thought this was not okay. But my superior behaved as if it was a correct thing to do, so did the PR. The PR even seemed to take charge of my superior. So I guessed this kind of practice was customary. (#5)*

Colleagues who were concerned about interactions with PRs encouraged three physicians' critical thinking (#3, 4, 6).

*One professor had a big influence on me. … One day, he told me that even small gifts like pens were problematic. I learned that he erased all logos inscribed on pens with a rasp. Since then, I have kept a rasp in my desk drawer. (#3)*

*I still remember a doctor who never used a taxi ticket offered by PRs, which I admired. I wonder why I admired his behavior. At that time only a minority of physicians did not use taxi tickets from PRs. (#4)*

*The department chief made clear his critical attitude toward the issue of conflict of interest. His attitude certainly had an influence on me. (#6)*

- Views of patients and the public

Awareness of patients' and the public's views on physician-PR interaction promoted critical attitudes toward gifts in four physicians (#1, 4, 5, 6).

*I had to be fair. If I was witnessed being bribed, that would be the end of me, I thought. (#1)*

*After I learned that some patients do not like physicians accepting even small gifts, I decided to remove from my pockets pens given by PRs. Now, I buy pens by myself. (#4)*

*When I was working in the public hospital, I noticed that the number of PRs visiting the hospital was gradually decreasing. That was in the late 1990's. I realized that the society's attitude toward medical profession became critical. (#5)*

*I don't mind using a pen given by PRs, but I do mind being seen using it by others. I'm afraid that some patients who are conscious of physician-industry relationships might consider me insensitive to the issues if I use pens given as gifts by PRs. (#6)*

- Acquisition of skills in information seeking and EBM

The internet clearly transformed physicians' information seeking skills. Four physicians (#1, 3, 4, 6) became less dependent on PRs after the emergence of the internet and two (#1, 5) became more critical about information given by PRs after learning about EBM.

In the old days, we didn't have the internet. A library was accessible only when you were working in a university hospital. We asked PRs to obtain articles. I think it cost as much as thousands of yen for one. (#4)

I do not obtain information from PRs at all. Electronic mailing lists helped me very much. Any information is available on the internet. (#6)

I regularly attended study sessions on EBM for residents at one teaching hospital. As I studied EBM, I became convinced of the concepts of EBM and of its importance in clinical practice. I became capable of obtaining information and critically appraising it by myself, without help from PRs. When information became ubiquitous on the internet, I did not need PRs. (#1)

- Learning about the concepts of professionalism and conflict of interest

Three physicians (#2, 6, 9) became more aware of the issues after learning about the concepts of professionalism and conflict of interest.

*When I went to an academic meeting, I noticed the term “COI (conflict of interest)” on posters. I became interested and looked into the issue after that. (#2)*

*Partly due to the influence of my supervisor then, I became conscious about medical professionalism. I heard someone saying that professionalism is an essential component of postgraduate education. I wondered what “professionalism” meant and started studying it by myself. (#6)*

*I bought a book for medical teachers. One of the chapters of the book dealt with professionalism and gift-relationships. I read it and learned a lot. I became more interested in learning about professionalism. (#9)*

Concerns that promotion could compromise their ability to prescribe rationally made five physicians (#1, 2, 3, 8, 9) think about keeping away from interacting with PRs.

*I read a report saying that even a pen, if there was a logo on it, could influence a physicians' prescribing behavior. I came to think that receiving even such small gifts was not right. (#1)*

*Although I'm relatively immune to influence, if I was specially treated by a company, it is possible that I could change my prescribing. I think, after all, that I am prone to be influenced by gifts. I was always thinking that I should keep away from such gift-relationships. (#2)*

*After we listened to a presentation by PRs about one drug, dishes of blowfish were served. I thought that this promotional activity was too costly; I wondered if it could hinder our correct judgments. (#3)*

*I liked to choose the medication for my patients in an unbiased way. I believe that I am the last person to be influenced. At the same time, I am afraid of being affected by promotions if I attend an industry-sponsored seminar. (#8)*

### Stage 3: Change in Behavior (Action)

The change in attitude was not necessarily followed by behavioral change, apparently due to rationalization and conformity to social norms.

- Rationalization

Even four physicians (#2, 5, 8, 9) who were aware of the potential for ethical problems in interaction with PRs rationalized their continuing relationships with PRs emphasizing the benefits.

*There are many pharmaceutical companies and not all PRs are the same. Some PRs bring really useful information. We should not dismiss all of them. Of course, we have to be careful not to go too far with them…. We should establish more professional relationships in which PRs offer valuable information to us and we critically appraise that information by ourselves. (#2)*

*Perhaps as an excuse for myself, I think the industry-sponsored seminars improve my clinical practice and benefit my patients. It is OK. Depending on time and circumstances; I only listen to a lecture and go home without attending the “happy hour” after the lecture. (#8)*

*Some PRs are seriously scientific. If I rejected all PRs, I would lose opportunities to learn useful information. In fact, PRs who are pharmacists have much more knowledge about pharmaceuticals than we do. That's why I don't reject all PRs. I don't listen to PRs who only say the names of their medications repeatedly. (#8)*

- Conformity to social norms

Four physicians (#1, 2, 3, 8) were sometimes in situations where they had to go along with their groups even if they didn't believe in what the group requested.

*When I was the director of the hospital, I requested PRs to stop serving lunch boxes at the industry-sponsored seminars for pharmaceutical medications. After a while, some doctors started to complain that it was normal to have lunch served during the seminars. I asked the food and nutrition service in the hospital to prepare sandwiches for the seminar. But it did not last long, partly because it was extra work for staff at the food and nutrition service. After all, we had to rely on the industry for lunch boxes at the seminars. (#1)*

*As the chief of a department, I could strictly regulate relationships with PRs. But, I'm afraid that strict regulation may force many colleagues to leave our department, like one of the proverbs, “clear water breeds no fish.” I wouldn't like to get our priorities backwards. For now, I hope that they are at least aware of the issues of physician-industry relationships and their ethical implications. (#3)*

*At the industry-sponsored seminars, I sometimes join “happy hour” after lectures. At the seminars, I see doctors who taught me a lot when I was young. They invite me to join “happy hour”. I don't want to reject their request, even though I am honestly not willing to join them because of my status as the director of a public institution. (#8)*

## Discussion

In the early years of their career, the interactions with PRs were passive. They were unaware of the implications of such interactions. As their career progressed, they started to think more actively about how they should interact with PRs and their attitudes toward PRs changed over time. The attitudinal changes were influenced by a variety of factors. Two thirds of our participants often had difficulty in making their behaviors consistent with their attitudes.

All the participants had interaction with PRs in the same manner as their superiors did in their early years and began to actively think about their interactions with PRs thereafter. This process is consistent with the findings of the qualitative report of how residents learn in the clinical workplace, in which residents were shown to act first and after that interpret the meaning of the activities [39]. This learning process can also be explained by the theory of situated leaning, in which learners enter the community of practice at the periphery and they move towards fuller participation by absorbing and being absorbed into the culture of practice [40]. As their career progresses, they started to have their own attitude toward relationships with PRs. In our study, their attitudes proved to be not constant but changing due to a variety of factors, including work environment, role models, views of patient and the public, acquisition of skills in information seeking and EBM, and learning about the concepts of professionalism and conflict of interest.

Transitions into teaching positions provoked ethical consideration about physician-PR relationships in some participants, which is consistent with the results of recent surveys. In a survey of residents and faculty members at a US medical school, faculty members was shown to be more concerned about direct contact between residents and PRs than residents themselves [41]. In the survey of physicians across the specialties, attending physicians had a more critical attitude toward receiving gifts from PRs than non-attending physicians [42]. We speculate that attending physicians may have a more critical attitude toward the interactions because they believe that residents and medical students are more vulnerable to influence from such interactions than themselves.

Some became hesitant to form relationships with PRs when they recognized views of patients and the public on the interaction. They were probably afraid of losing patients' and the public's trust. Patients' trust in physicians was shown to be affected by gifts from the industry to physicians in recent surveys [8], [9]. On the other hand, most physicians in the US agreed to the statement,” physicians should put the patient's welfare above the physician's financial interest [43].” Thus, announcing the results of the patient surveys to physicians is expected to promote their critical attitude toward the relationships. In 1993, to conform to the revised rules of Japan Fair Trade Commission, Japanese Pharmaceutical Manufacturers Association established the first version of Promotion Code for Prescription Drugs which restricted industry support to physicians unrelated to the products to 100,000 Japanese Yen (US $800.00) per institution [23]. The Code substantially changed the way the pharmaceutical representatives interact with physicians. It is likely that such changes in the society made some physicians feel that the views of the public toward physician-PR relationships became more critical.

Some participants indicated that fear of negative influence from gifts on their own prescribing behaviors affected their attitude toward relationships with PRs. The survey of psychiatry residents, intern and clerks in the University of Toronto showed that there was a positive correlation between the number of promotional items received and the belief that discussions with representatives have no impact on prescribing behavior [44]. In other words, enhancing perceived influence by interaction with PRs will possibly reduce the interaction. Also, evidence of the influence of promotion on physicians' behavior will be an important and effective component of teaching physician-PR relationships.

However, physicians' attitudinal changes affected by a variety of events did not necessarily lead to behavioral changes. Participants often legitimatized and rationalized their continuing interactions with PRs even when they understood that such interactions might have unfavorable effects on patient care. This is compatible with the results in recent qualitative studies, which showed that physicians used rationalizations to resolve their cognitive dissonance [31], [45]. The other factor contributing to the inconsistency between attitude and behavior is considered to be conformity to social or cultural norms [31], [32]. “Social norm” is defined as the implicit rules for acceptable behaviors, values, and beliefs in social psychology. We often get along with a group even if we don't believe they are right and behave as if we accept them, which is explained as public compliance without private acceptance under normative pressure [46]. According to the results of our study, they seemed to know their group's behavior was ethically problematic but they were pressured to conform to the group's norms. Inversely, using the tendency to conform to social norm can be effective in promoting beneficial behavior [46].

One of nine physicians (#6) worked in the hospital where he was not allowed to have any interaction with PRs. He was the only “avoider” among the participants. The likely reason why he was an “avoider” is early abstention and being kept in a work environment without physician-PR relationship. This case supports the notion that creating new social norms that disapprove physician-PR relationships may help individual physicians to avoid such relationships.

Our study has limitations. First, participants were hardly typical of Japanese physicians because they all attended the symposium of conflict of interest with strong interest, which may limit transferability of the findings. However, we believe that having some knowledge about the concept of conflict of interest helped them to reflect on their attitude and behavior. Second, social desirability bias and post hoc rationalization may have concealed some negative aspects of their attitude and behavior. Third, the accuracy of our participants' stories especially with regard to changes in their attitudes and behaviors may not correct, because their stories were constructed backwards based on their memories at the time of the interviews. It is possible that participants would have revealed different stories if we had repeated interviews over time. Fourth, coders' bias in the data analysis in qualitative studies cannot be totally avoided. We attempted to minimize such bias by having the whole process from coding to theorization visualized on Excel file and having it reviewed by three authors.

In conclusion, physicians' attitude toward relationships with PRs changed over time and a variety of factors affected such change. Paying attention to these factors and creating new social norms may be both necessary to produce change in behavior consistent with change in attitude.
